# An observational study protocol on wastewater measurements and diaper culture to estimate antimicrobial resistance in long-term care facilities for people with intellectual disabilities: The GIRAF-MIC study protocol

**DOI:** 10.1371/journal.pone.0324083

**Published:** 2025-05-15

**Authors:** S. Hidad, S.C. de Greeff, F. de Haan, R. Schilperoort, G.L. Leusink, A. Timen, H. Schmitt

**Affiliations:** 1 Centre for Infectious Disease Control, National Institute for Public Health and the Environment, Bilthoven, the Netherlands; 2 Department of Primary and Community Care, Radboud University Medical Center, Nijmegen, the Netherlands; 3 Partners4UrbanWater, Nijmegen, the Netherlands; Universidad San Francisco de Quito, ECUADOR

## Abstract

**Introduction:**

Antimicrobial resistance (AMR) has become one of the leading global health threats. It is critical to understand the burden of AMR, particularly among vulnerable populations such as people with intellectual disabilities residing in long-term care facilities (ID-LTCFs). Traditional study methods to estimate the burden of AMR in these settings, such as rectal swabs to measure the prevalence of MDRO carriage, are considered burdensome for this population. This underscores the importance of a non-invasive method to assess the burden of AMR among people living in ID-LTCFs. This publication describes a study protocol for an alternative approach to estimate AMR, specifically Extended Spectrum Beta-Lactamase (ESBL) and carbapenemase-producing Enterobacterales (CPE), in ID-LTCFs in the Netherlands, through wastewater measurements combined with analysis of stool collected from diaper material. The protocol provides detailed information about the study design and methodologies proposed for a pilot study.

**Methods and analysis:**

Wastewater samples will be obtained from the sewers of ID-LCTFs using passive samplers. Additionally, as a considerable part of ID-LTCF residents are incontinent, stool samples will be collected from diaper material which will be obtained from incontinent residents living in participating ID-LTCFs. The wastewater and stool samples will be cultured on selective media to detect ESBL-producing Enterobacterales and carbapenem producing Enterobacterales (CPE) strains. Determination of strains will be carried out using MALDI-TOF and phenotypical tests will be carried out to confirm ESBL and CPE producing strains. In wastewater samples, bacterial concentrations will be determined, expressed in colony forming unit (CFU) per passive sampler, while in stool from diaper material the presence or absence of ESBL and CPE will be reported in proportions.

**Ethics and dissemination:**

The procedures described in this study protocol will be conducted in line with principles outlined in the Declaration of Helsinki, Code of Conduct for Health research, as well as the General Data Protection Regulation. Approval in advance by an ethical research committee or institutional review board is deemed unnecessary by current national and European legislation.

## 1 Introduction

Antimicrobial resistance (AMR) has become a leading global public health threat according to the World Health Organization with an estimated 1.27 million AMR attributable deaths per year [1,2].

In public health, several factors contribute to AMR but the most important ones are the overuse and misuse of antibiotics in humans and animals, and the poor implementation of infection prevention and control measures. The interplay of these factors contributes to the development and spread of resistant microorganisms which reduces the efficacy of treatment with antimicrobials, and poses a serious risk for global health [3].

Wastewater based epidemiology (WBE) is increasingly used for surveillance of public health threats [4–7]. WBE has been used to monitor viral pathogens such as poliovirus, hepatitis viruses, viral gastroenteritis and SARS-CoV-2 [6–9]. In recent years, it has been employed to track bacterial microorganisms including multi-drug resistant organisms (MDROs) [9–13]. This approach is particularly valuable when traditional AMR surveillance methods, which rely on individual microbiological diagnostics, are difficult to apply. A specific group which is often overlooked within public health initiatives and where routine methods are more difficult to apply are people with intellectual disabilities living in long-term care facilities (ID-LTCFs) [14]. This group is characterized by significant impairments originating from childhood, in both intellectual functioning as well as adaptive behavior, and bears a higher risk for chronic illness and comorbidities compared to people without ID [14–17]. Difficulties with sample collection for microbiological analysis or other laboratory tests due to behavioral problems and/or incontinence, may cause challenges in estimating the prevalence of MDRO carriage in such populations following traditional protocols. Given these challenges, it is necessary to explore tools that offer alternatives to currently existing AMR surveillance, which is based on clinical laboratory data [18].

One such tool consists of wastewater based analysis. The potential added value of wastewater measurements for detecting MDROs in ID-LTCFs lies in the noninvasiveness of this approach. However, it is important to note that approximately one-third of individuals with intellectual disabilities suffer from incontinence and might wear diaper material, meaning this group is not represented in wastewater measurements [19,20]. Therefore, in addition to wastewater measurements, we suggest collecting stool samples from diaper material.

In this study protocol, we provide detailed information about wastewater and diaper sampling methods to determine the extent of Extended-Spectrum Beta-Lactamase-producing (ESBL) Enterobacterales and Carbapenemase-producing Enterobacterales (CPE) in ID-LTCFs in the Netherlands. This study protocol includes details on the study design and sample collection procedures, and describes strengths and limitations of the methods used. The study protocol will be used for a pilot study to further evaluate the applicability of this approaches in practice. This paper does not include any study findings or results.

## 2 Methods and analysis

### 2.1 Study design, recruitment and inclusion of care facilities

Data will be collected cross-sectionally using three study instruments, namely: 1) passive samplers for wastewater sample measurements, 2) stool samples from diaper materials and 3) a short questionnaire to collect demographics of participating ID-LTCFs.

In this study protocol, the aim is to: I) conduct three sewer measurements per location and/or II) collect a minimum of five stool samples from diaper materials per location, from unique residents. The minimum of five stool samples is intended to prevent traceability to participating individuals.

Before the start of the sample collection, all organizations interested in participation receive a comprehensive information package detailing participation in the study. This package outlines the study protocol, data collection procedures, data processing methods, information on the measures taken to ensure the privacy of participants (incontinence), and the potential benefits and drawbacks of participation for the organization and the residents in participating ID-LCTFs.

After obtaining approval for participation from the care organization or facility, the sewer system will be inspected to locate an appropriate measurement point for wastewater sample collection. The goal is to find a sampling site which accurately reflects a significant portion of the residents living in ID-LTCFs with no contribution of wastewater from additional surrounding areas or buildings (adjacent houses, other care facilities within the same building). This will be done by inspecting the map of the participating facility and the surrounding municipal sewer lines, which will be collected from the municipality and facility itself. In addition, inspections around the facility will be conducted to confirm appropriateness of the existing maps (as maps can be outdated). In some cases, additional tests may be conducted to confirm the most appropriate measurement location within the sewage system for the most optimal wastewater sample collection, consisting of releasing food colouring agents (dye testing) into facility toilets and visually monitoring colour signals at the intended measurement points. To install the wastewater samplers, it is essential that the measurement point in the sewage pipe is easily accessible.

Participation in the study is possible when ID-LTCFs meet the following inclusion criteria:

- Demonstrated interest and consent on participation from local scientific commissions or board;
- and availability of one dedicated local study coordinator within ID-LTCF during the study period;
- and suitable sewer system for wastewater measurements;
- or interest in participating in diaper study only when sewer system is not appropriate for wastewater measurements;

### 2.2 Sample collection

#### 2.2.1 *Wastewater sampling using passive samplers (water samples).*

**Fig 1:**
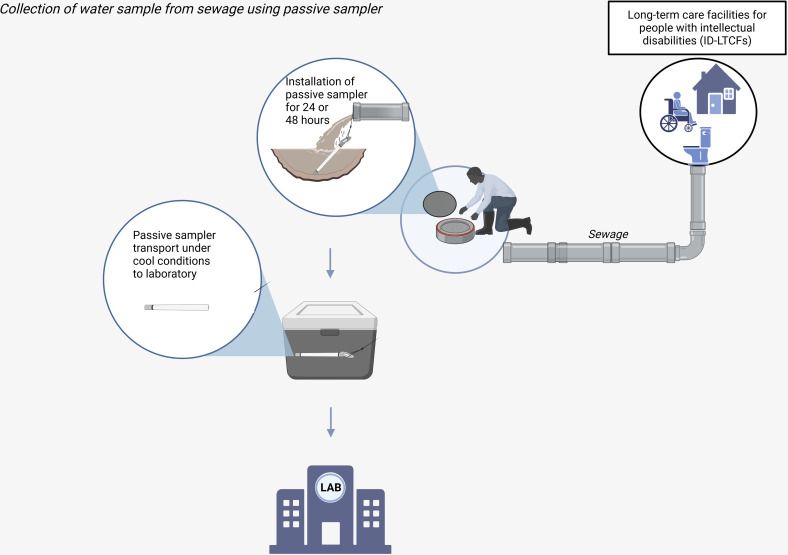
Collection of water samples from sewage using passive sampler. Created in BioRender. Hidad, S. (2025) https://BioRender.com/hz5f16k.

For collection of wastewater samples we will make use of passive sampling. Passive sampling is characterized by transfer of microorganisms between the sampled medium, like wastewater, and a collecting medium (cotton tip and gauze), commonly known as a passive sampler [21]. The widely used “Moore swab” technique employs cotton gauze suspended in water to gradually accumulate micro-organisms. This has been previously described in studies to detect enteric bacteria and viruses such as polio and human norovirus [22–24]. The use of passive samplers represents an alternative cost-effective approach to collect composite wastewater samples compared to conventional autosamplers [25–27], and offers the potential to sample more representatively as compared to single grab samples. For the wastewater sample we will use a “torpedo” sampler which contains cotton tips and gauze in a plastic casing to gradually accumulate microorganisms [28]. The torpedo sampler will be installed at a specific measurement point around the ID-LTCFs to only capture wastewater from this participating ID-LTCF. Three independent sampling events are planned per participating ID-LTCFs in a timespan of maximum ten days, between October 2024 and June 2025. Three sampling events are deemed necessary to increase the likelihood of capturing defecation events of all residents, given that the frequency of defecation can be smaller than 1 time a day [29] and constipation is highly prevalent among institutionalized people with ID [30], further reducing frequency of defecation. These three events can be achieved within a time span of 10 days. We aim to collect water samples within 9 months, avoiding August and September, for all participating ID-LTCFs in order to reduce possible seasonal effects on ESBL carriage [31]. After installment, the passive sampler remains in place for a duration of 24 or 48 hours, in order to capture the longest deployment time over the 10 total days possible within the boundaries of laboratory sample analysis (requiring two consecutive days). The deployment scheme consists of a first 48 h measurement (mon-wed), followed by a 24h measurement (wed-thu) and a second 48h measurement (mon-wed), in total capturing 5 out of the 10 days in total. Subsequently, the passive sampler will be removed and transferred into a plastic bag whereafter the passive sampler is transport in cooled conditions (maintained between 4–8 degrees Celsius). Laboratory procedures for analysis are conducted within 24 hours after passive sampler collection, preferably on the same day [32]. In Fig 1, the collection of water samples is illustrated.

For the wastewater measurement there is no individual consent or opt-out needed from residents living or working within the participating ID-LTCFs as there is no ‘trace-back’ possible to individual persons from wastewater.

#### 2.2.2 *Sampling of diaper material (stool samples).*

Each ID-LTCF is asked to organize an opt-out procedure to inform their residents about the study and allowing residents and their legal representatives the opportunity to raise objections to participation. In the event of objection, diaper material from these residents will not be submitted for the study. In the case that no objection is raised, it is the participating ID-LTCF responsibility to collect the diaper material (or stool sample). Therefore ID-LTCFs are requested to organize an procedure in which they record opt-out requests in an appropriate manner, such as in electronic medical records of those who do not want to participate in the study. This only applies to the collection of diaper material and does not apply to opt-out for wastewater measurements.

Diaper material will be collected without personal data of the resident. There is no maximum number of diaper materials that is eligible for inclusion. However, a minimum of five diapers is required to prevent traceability to participating individuals. Diapers will be included when: 1) the pad is saturated with visible stool, and 2) the diaper or stool from diaper is stored under cool conditions for a maximum of 24 hours. Transport and laboratory procedures for analysis are conducted within 24 hours after diaper collection, but preferably on the day of arrival at the laboratory [32], such that the maximum time between diaper collection from the participant and laboratory assessment in the laboratory is 48 hours.

#### 2.2.3 Questionnaire.

A web-based questionnaire will be used to collect general characteristics of the participating ID-LTCFs and, on an aggregated level, of its residents (see Table 1). These background variables will be used to interpret microbiological findings from the wastewater and diaper analysis.

**Table 1:** Questionnaire to assess general characteristics of ID-LTCFs.

Questionnaire
What is the name of your organization?
What is the name of the participating location?
What is the address of the participating location?
Under which GGD (regional health service) does your location fall?
How many people reside at the location?
How many residents/ which proportion with incontinence material are at the participating location?
Are there doctors and nurses working within the institution? If so, can you briefly describe how this is organized? (intellectual disability physician, GP on/off-site, nurses on/off-site)
What client target group resides at the participating location? Check all that apply. You can select more than one option: Mild intellectual disability Moderate intellectual disability Severe multiple disabilities Very profound intellectual and multiple disabilities
Are facilities such as sanitation shared by residents at this location?
Can you indicate how many known ESBL-infected individuals or carriers you have within the participating location, if possible?
Can you indicate how many known CPE-infected individuals or carriers you have within the participating location, if possible?

### 2.3 Bacteriological culture process

#### 2.3.1 Culture media.

Culture based techniques will be carried out to identify and quantify ESBL and CPE in both water and stool samples as outlined below. In addition, selective culture of *E. coli* on Tryptone Bile X-glucuronide (TBX agar) from Biorad is undertaken. The ChromID™ ESBL (bioMérieux, Marcy-l’Étoile, France) medium (from here on termed ESBL medium) enables selective growth of ESBL-producing *Enterobacterales (*pink to burgundy coloured colonies for *E. coli* and green to blue coloured colonies for *Klebsiella, Enterobacter, Serratia* and *Citrobacter* strains (KESC) based on β-glucosidase activity). The ChromID™ OXA (bioMérieux, Marcy-l’Étoile, France) and ChromID™ CARBA (bioMérieux, Marcy-l’Étoile, France) media (from here on termed OXA and CARBA media) will be used to detect *Enterobacterales* producing OXA-48-like carbapenemases, metallo-beta-lactamases (e.g., NDM, IMP, VIM etc.) and class A Serine type carbapenemases (e.g., KPC).

#### 2.3.2 Microbiological analysis of water samples.

After arrival at the laboratory, the samplers are processed by opening the casing and removing the collecting medium (cotton tip and gauze).

Bacteria are desorbed from collecting medium (cotton tip and gauze) by vortexing in 10 mL of preservation solution (physiological salt solution) in 50 mL tubes. To ensure thorough mixing, the tubes will be vortexed for a minimum of 1 minute. Following this, a decimal dilution series is prepared. Appropriate decimal dilutions for each individual collecting medium (cotton tip and gauze) will be filtered through 0.45μm pore size membrane filters following general microbiological procedures for water samples [33]. ESBL analysis will be based on cotton tips (as these showed better linearity of their calibrations against wastewater with known concentrations of *E. coli* and ESBL than gauze, unpublished results) while gauze will be used to detect CPE (as gauze accumulates more microorganisms per passive sampler and is therefore more sensitive to detecting bacteria with an assumed low concentration [26,28,34].

The filtered volumes for *cotton tips* are equivalent to 0,003ml, 0,001ml, 0,0003ml, 0,0001ml of the original sample for TBX agar, and 0,3ml, 0,1ml, 0,03ml and 0,01ml for selective ESBL agar.

The filtered volumes for *gauze* are equivalent 7.5mL, 2mL, 0,1 and 0,01mL for CARBA and OXA agar.

**Fig 2:**
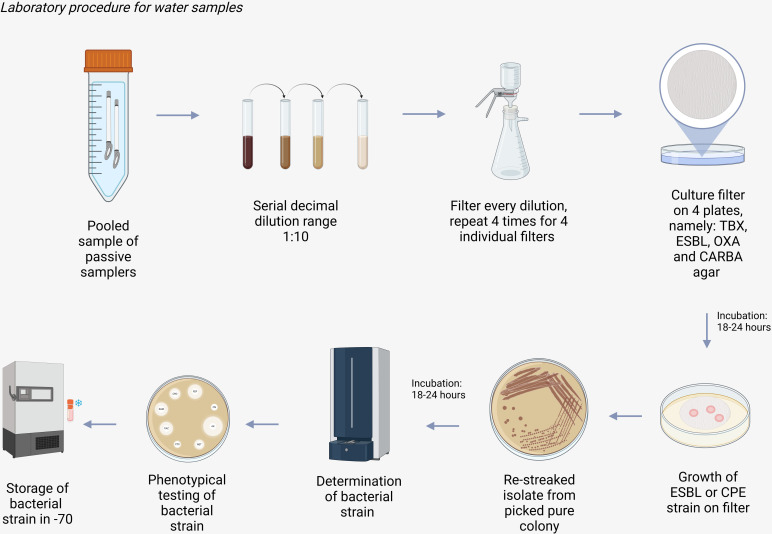
Laboratory procedure for water samples. Created in BioRender. Hidad, S. (2025) https://BioRender.com/c86hv82.

After filtration, the filters are placed on the designated culture plate (TBX, ESBL, CARBA or OXA agar) and incubated for 4–5 hours at 36 °C ± 1 ˚C followed by 18–20 hours at 44°C ± 1 ˚C. Incubation at 44°C ± 1 is necessary to inhibit growth of other background bacteria [9]. In Fig 2, the laboratory procedure for water samples is illustrated.

#### 2.3.3 *Microbiological analysis of stool samples from diaper materials.*

Prior to culture, 1g + - 10% of the feces will be collected from the diaper material using a sterile cotton swab or spatula and transferred to Buffered Peptone Water (BPW) medium for enrichment (in a 1:10 dilution). This enrichment is incubated for 18–24 hours at 36 ± 1°C. The remaining feces will be directly inoculated using the 4 quadrant method on selective agars: ESBL, CARBA and OXA plates and incubated in ambient air at 36 ± 1°C for 18–24 hours [9].

#### 2.3.4 *Strain selection, determination and phenotypic testing.*

For presumptive ESBL producing isolates growing on ESBL agar plates, we will select two *E. coli* colonies and 5 non-*E. coli Enterobacterales* colonies for subculture on Tryptic soy agar (TSA) agar followed by incubation at 36 ± 1 ˚C for 18–24 hours, in preparation for MALDI-TOF determination and phenotypic testing.

All presumptive CPE producing isolates growing on CARBA or OXA agar plates will be subcultured, first on CARBA or OXA agar plates, depending on which plate colony was observed, and subsequently on TSA agar plates, followed by incubation at 36 ± 1 ˚C for 18–24 hours, in preparation for MALDI-TOF determination and phenotypic testing.

Re-streaked isolates from TSA agar plates will be maintained in MicrobankTM vials (Pro-Lab Diagnostics) and prior to storage confirmed using MALDI-TOF MS.

**Fig 3:**
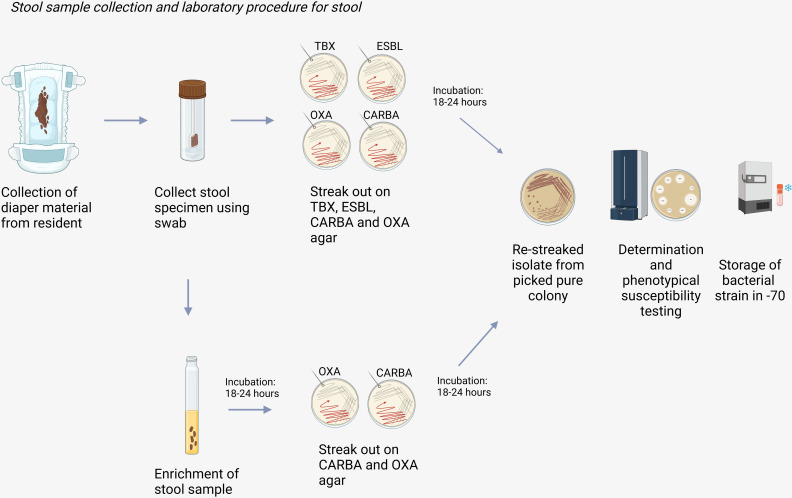
Collection of stool sample and laboratory procedure for stool. Created in BioRender. Hidad, S. (2025) https://BioRender.com/inpk1ts.

Phenotypic testing will be done following National microbiological guidelines for ESBL phenotypical confirmation [35], and following Howard et al. 2020 for confirmation of carbapenemase production by in-house Carbapenem inactivation method iCIM [36]. In Fig 3, collection of stool from diaper material and the laboratory procedure for stool samples is illustrated.

#### 2.3.5 *Quality control.*

Positive and negative (physiological salt, buffered peptone water) quality controls will be carried out for all agar plates and agents used.

Agars will be tested qualitatively by inoculation with positive and negative control strains (Table 2).

**Table 2:** Positive and negative quality control strains per agar plate.

Medium	Positive control strains	Negative control strains
**TBX**	*E. coli* ATCC 25922	
**ChromID ESBL**	*ESBL E.coli EE 17-0069-2* (resistance genes CTX-M9; CTX-M14 or CTX-M17)	*E. coli* ATCC 25922
**ChromID OXA48**	CPE *E.coli* 30112023 RWS (resistance genes OXA-48 and NDM)	*E. coli* ATCC 25922
**ChromID CARBA**		

Quantitative microbial controls will be included during filtration of water sample using quantitative *E. coli* controls. Quantitative results will be discarded for experiments when the control value exceeds average ± 3x standard deviation (SD).

For testing sterility of passive samplers, agars, filters and other equipment used during the filter procedure, 10mL PS will be filtered for every agar type concurrently with the filters used for water samples. In cases where contamination is detected, whether with the target bacteria or a significant contamination with another species, the results must be invalidated, regardless of which of the three components was contaminated. Additionally, each new batch of membrane filters undergoes testing against the previous reference lot [37].

### 2.4 Sample size calculation

#### 2.4.1 *Wastewater measurements.*

A sample size calculation for the number of wastewater samples required to detect an elevated concentration above the population baseline is not possible due to the lack of data on the variation of fecal concentrations for ESBL between individual carriers. For this reason, within this study protocol we aimed to include at least five different ID-LTCFs geographically distributed throughout the Netherlands.

#### 2.4.2 Diaper material (stool samples).

The sample size calculation for stool samples from diaper materials, we assumed a population size of 67.000 (e.g., number of people with intellectual disabilities in the Netherlands receiving care under the Long-term care act (WLZ). From this population, around 26% is estimated to have incontinence (and wear diaper materials) [20]. The ESBL carriage prevalence in the general population in the Netherlands is approximately 4.5% [38]. With a 5% error and 95% confidence level, the required sample size for diaper materials would be 67 incontinent individuals living in ID-LTCFs.

### 2.5 Data management

Throughout the study we will collect and secure personal contact information of local study coordinator and the communication history (letters, emails, and phone calls) in a password-protected database, kept separate from questionnaire and sample data, and access is restricted to study team members only. Moreover, all anonymously collected laboratory data are documented in an protected environment which is only accessible by members of the research group. Moreover, adhering to FAIR principles, we ensure that our data management practices facilitate Findability, Accessibility, Interoperability, and Reusability [39]. This includes storing research data in publicly accessible and easily discoverable repositories.

### 2.6 Data-analysis

In water samples, the primary outcome variables include the concentration of ESBL in colony forming units (CFU) per passive sampler. For ESBL, as community prevalence is around 5% [38], presence of single carriers even in relatively small populations is possible. Therefore, analysis aims at quantitative comparisons between institutions, and if possible, with the overall Dutch community.

For ESBL, the analysis aims at quantitative comparisons between the participating ID-LTCFs. The main outcome variable is the number of ESBL detected per sampling event of 24 hours in the passive samples. Additionally, if possible, these results will be converted to quantitative data comparable with the overall Dutch community. To enable this, ESBL concentrations per passive sampler will be transformed into the concentration of ESBL per milliliter of wastewater using previously established calibrations of concentrations of *E. coli* in wastewater as compared to passive sampler collecting media determined in wastewater treatment plants (Schmitt et al., unpublished results). The estimated load of ESBL in wastewater is expressed as the total number of bacteria per resident at each participating ID-LTCF. This calculation necessitates data on the number of residents (neglecting staff/ visitor numbers) and estimations of the water consumption in the sampled ID-LTCFs (site). If sufficient reliable data or estimates of water consumption in the measured section of the site are available, these ESBL loads expressed in numbers of ESBL per person can be determined and compared with ESBL loads in the general population [40].

Based on growth of *E. coli* on the TBX and ESBL agar plates we will assess the non-ESBL producing *E. coli* versus ESBL producing *E. coli* ratio. This can be achieved by counting the colonies on TBX (non-selective agar plate) and ESBL plate [41].

For CPE, data is reported as presence/ absence in wastewater per ID-LTCFs, as the community prevalence of CPE is so small [38] that the potential presence of a single carrier among the residents is deemed a relevant outcome.

In diaper materials, the primary outcome variables in this study will be the percentage of ESBL and/or CPE positive samples. The percentage of ESBL and/or CPE will be calculated by dividing the number of positive diapers (stool samples) containing ESBL and/or CPE by the total number of analyzed diapers per ID-LTCF and for all participating ID-LTCFs.

#### 3.6.1 *Post-hoc analysis: Sequencing.*

Depending on the findings, a decision will be made during the project on whether a selection of bacterial strains will be further characterized using whole genome sequencing. The aim is to map the genetic information of the identified organism and compare genetics of isolated strains. The relatedness of isolated strains can be studied between strains from stool samples of an ID-LTCF as well as between strains collected from water samples and stool samples within the concerning LTCF.

## 3 Ethics and dissemination

### 3.1 *Ethical consideration*

Approval by an ethical research committee or institutional review board was considered unnecessary under current national legislation. This decision is based on the study meeting two criteria as outlined in Article 1 of the Dutch WMO; 1) there is no involvement in medical-scientific research and/or subjecting individuals to burdensome procedures or imposing specific behaviors on them as part of the study protocol, 2) the study does not involve a clinical trial with medicinal products for human use. The research will be conducted by the principles outlined in the Declaration of Helsinki and the Code of Conduct for Health Research, as well as the General Data Protection Regulation (GDPR).

### 3.2 Patient and public involvement statement

We have established an advisory panel which consists of multi-disciplinary professionals working in the field of intellectual disability care, epidemiology, infection prevention and control, and microbiology. Moreover, members of the research group are affiliated to the Academic Collaborative Center “Stronger on your own Feet”, where collaboration with co-researchers (researcher with intellectual disability) or (representatives from) patients will be facilitated if needed [42]. During the study, we will consult the advisory panel or co-researchers with any dilemma or challenge faced when conducting the study in ID-LTCFs. This panel will also facilitate dissemination of study results within their respective discipline or sector.

### 3.3 Reporting back to ID-LTCF

For CPE, data is ultimately reported back to participating ID-LTCFs as presence/ absence per participating ID-LTCF. For ESBL, data is ultimately reported back to participating ID-LTCFs in percentages if the proposed sample size is reached, otherwise limited to the presence/absence of ESBL.

Findings from the to-be-conducted study will be published in a peer reviewed journal and widely disseminated among participating ID-LTCFs and within the Dutch disability sector and other relevant stakeholders.

## 4 Discussion

Currently, there is a lack of data on the estimates of ESBL and CPE among people with intellectual disabilities. Here we present a non-invasive method to estimate the presence of ESBL and CPE in populations where classical methods such as rectal sampling to identify carriage of MDROs are less feasible due to the nature of the population. The proposed approach as described in this study protocol aims to provide a first insight into the magnitude of ESBL and CPE among institutionalized people with ID in the Netherlands. With regard to the proposed research methods, some limitations need to be acknowledged. First, it is important to note that capturing MDROs by wastewater measurements in ID-LTCFs does not allow identification of individual persons carrying MDROs, and the estimate cannot exclusively be attributed to people living in long-term care facilities. Others, including (healthcare) professionals, and visitors can also contribute to the ESBL and/or CPE in wastewater. This also means that quantitative measurements of ESBL per passive sampler and ESBL per resident, evaluated in order to compare between different ID-LTCF and/or between ID-LTCF and the general population, will be influenced by possible contributions of professionals and visitors, although visitors are less likely to defecate during visits of short duration. Furthermore, not all ID-LTCFs will likely offer the possibility to identify single suitable sewer sampling sites without influence of other buildings, which will then be excluded for participation. Second, due to the highly variable concentrations of ESBL (and presumably CPE) in human stool [43], we are unable to precisely estimate the number of potential carriers based on wastewater measurements. Finally, we are unable to validate the performance of the passive sampler against ‘above ground’ data as we only have access to stool from diaper wearing residents thus miss stool samples directly collected from residents not wearing diapers. Findings from water and diaper samples therefore should be interpreted separately, however it might be expected that if the prevalence of ESBL and/or CPE is relatively high in both samples, this might indicate transmission within the ID-LTCF.

Although the findings that will be accessible through this protocol should be interpreted with caution, the proposed approach has also several strengths. First, this study will shed some light on a group within society that is underrepresented in routine microbiological data (due to limited symptom articulation and barriers in sample taking) and not identifiable in routine surveillance data as the intellectual disability is often not mentioned in patient records available at the laboratory. Second, we will use surveillance of AMR in wastewater as a population-representative sample, as has been suggested for influent from wastewater treatment works (WWTW) [44,45]. Third, the approach is considered as minimal invasive to people with ID, as we will only slightly interfere with daily and routine care received by the residents in ID-LCTs. In conclusion, the combined effort to explore AMR in wastewater and in stool from diaper materials among incontinent residents will provide a multi-angle insight – also considering those who are not always captured following standardized protocols.
